# Detoxification of Aflatoxin B_1_ by *Zygosaccharomyces rouxii* with Solid State Fermentation in Peanut Meal

**DOI:** 10.3390/toxins9010042

**Published:** 2017-01-20

**Authors:** Guanghui Zhou, Yujie Chen, Qing Kong, Yunxiao Ma, Yang Liu

**Affiliations:** 1 School of Food Science and Engineering, Ocean University of China, Qingdao 266003, China; 17864272801@163.com (G.Z.); 13361285003@163.com (Y.C.); 15684737858@163.com (Y.M.); 2 Institute of Food Science and Technology, Chinese Academy of Agricultural Sciences/Key Laboratory of Agro-Products Processing, Ministry of Agriculture, Beijing 100081, China

**Keywords:** aflatoxin B_1_, detoxification, *Zygosaccharomyces rouxii*, solid state fermentation, LC/MS

## Abstract

Aflatoxins are highly carcinogenic, teratogenetic, and morbigenous secondary metabolites of *Aspergillus flavus* and *A. parasiticus* that can contaminate multiple staple foods, such as peanut, maize, and tree nuts. In this study, *Zygosaccharomyces rouxii* was screened out and identified from fermented soy paste—one kind of traditional Chinese food—to detoxify aflatoxin B_1_ (AFB_1_) by aerobic solid state fermentation in peanut meal. The optimal degradation condition was chosen from single factor experiment, and the most effective detoxification rate was about 97%. As for liquid fermentation, we tested the binding ability of *Z. rouxii*, and the highest binding rate reached was 74.3% (nonviable cells of *Z. rouxii*) in phosphate-buffered saline (PBS). Moreover, the biotransformation of AFB_1_ through fermentation of *Z. rouxii* in peanut meal was further verified by liquid chromatography/mass spectrometry (LC/MS). According to TIC scan, after fermentation by *Z. rouxii,* the AFB_1_ in peanut meal was prominently degraded to the lowering peaks of AFB_1_. Additionally, *m*/*s* statistics demonstrated that AFB_1_ may be degraded to some new products whose structural properties may be different from AFB_1_, or the degradation products may be dissolved in the aqueous phase rather than the organic phase. As far as we know, this is the first report indicating that the safe strain of *Z. rouxii* has the ability to detoxify AFB_1_.

## 1. Introduction

Aflatoxins—highly morbigenous secondary metabolites mostly generated by *Aspergillus flavus* and *A. parasiticus*—contaminate multiple staple foods, such as peanut, maize, and tree nuts, and cause a great loss to the economy and public health [1,2]. More than 20 types of aflatoxins have been found, and the most known and important ones are aflatoxin B_1_ (AFB_1_), aflatoxin B_2_ (AFB_2_), aflatoxin G_1_ (AFG_1_) and aflatoxin G_2_ (AFG_2_) [3]. AFB_1_ is the most poisonous one, and is associated with liver cancer, dysfunction of the immune system, and protein deficiency syndromes of humans and animals [4]. The key carcinogenic site of AFB_1_ is the double bond of terminal furan rings, and the main carcinogenic mechanism is to inhibit the synthesis of RNA [5]. So, it is imperative to reduce the concentration of aflatoxins in foods.

A series of strategies have been recommended for the management of aflatoxins in foods. Physical, chemical, and biological methods should comply with the following principles: (1) aflatoxins should be transformed to innocuous products; (2) foods or feeds should retain their nutritional value, and the material properties should not be changed enormously; and (3) it should be highly efficient [6]. So far, many physical and chemical methods had been used to eliminate aflatoxins, including treatment with gamma rays, ultraviolet (UV) light, heat, calcium hydroxide, hydrogen peroxide, chlorine gas, and so on [7,8,9,10,11]. However, most physical and chemical methods have limitations, such as losses of nutritional value and high cost [8,9,10]. Therefore, biological controls provide an eligible, attractive, and safe method to remove aflatoxins in foods [12,13].

Traditional Chinese fermented foods have existed for thousands of years and have been consumed by generations of Chinese people, and were conventionally fermented by natural microorganisms. Many studies have proven the safety of traditional Chinese fermented foods [14,15]. Traditional Chinese fermented foods are reservoirs of microorganisms, such as bacteria, yeasts, and moulds [16], but the microbial distribution and functions need to be further studied. For example, some new strains isolated from traditional Chinese fermented pork had an excellent capacity for nitrite-reduction, and one strain isolated from fermented dairy food could inhibit vascular tension and hypertension [17,18]. For this reason, the idea of screening out strains for the removal of fungal toxins from traditional Chinese fermented foods was extremely feasible [19,20].

Earlier, we combined heat treatment and anaerobic solid state fermentation of *Streptococcus thermophilus* and *Lactobacillus delbrueckii subsp. Bulgaricus* to detoxify aflatoxins in peanut meal [21]. *Saccharomyces cerevisiae* is the most well-known species of yeast, and has been studied to evaluate their ability to remove aflatoxins from liquid media [22]. The ability of *Bacillus megaterium* to significantly inhibit the growth of *A. flavus* and the biosynthesis of AFB_1_ was previously identified in our lab [23]. We hypothesized that these two strains may also remove aflatoxins in solid state fermentation. In this study, we successfully screened out one detoxification strain (*Z. rouxii*), and designed a single factor experiment to optimize the degradation conditions. The identification of degradation products of AFB_1_ by *Z. rouxii* was also performed by liquid chromatography/mass spectrometry (LC/MS).

## 2. Results

### 2.1. Screening Strains for the Detoxification of AFB_1_

Four yeasts and moulds were isolated from traditional Chinese foods, and the effect of different strains on the detoxification of AFB_1_ is presented in Table 1. AFB_1_ residual rates were significantly different in different strains (*p* < 0.01). The AFB_1_ residual rate of untreated sample was 100%, and the concentration of residual AFB_1_ was 115 μg/kg. After aerobic solid state fermentation, the residual rate of Strain B, Strain C, Strain D, *S. cerevisiae*, and *B. megaterium* groups had no conspicuous decrease. However, the AFB_1_ residual rate of Strain A and *S. thermophilus* groups was 16.18% and 17.63%, respectively, significantly lower than that of other groups (*p* < 0.01). Therefore, Strain A was selected for further identification and study.

### 2.2. Identification of Strain A

Firstly, the culture of Strain A was checked for purity and was given preliminary identification on the basis of their cell and colony morphologies and biochemical profiles. The cell of Strain A was round, and the diameter was 5 μm. The colony morphologies were milky white, smooth, moist, and shiny.

Secondly, the 26S rDNA gene of Strain A was amplified and sequenced. The resulting sequence (582 bp) showed that it had 100% similarity with 26S rRNA (JQ689016) of *Z. rouxii* strain NRRL Y-229. This result confirmed that Strain A was a strain of *Z. rouxii.*

### 2.3. Effect of the Heat Temperature and Heat Time on AFB_1_ Detoxification

**Figure 1:**
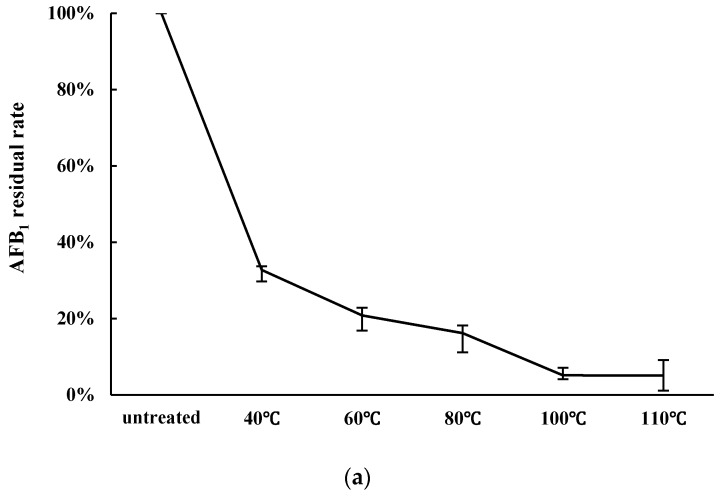

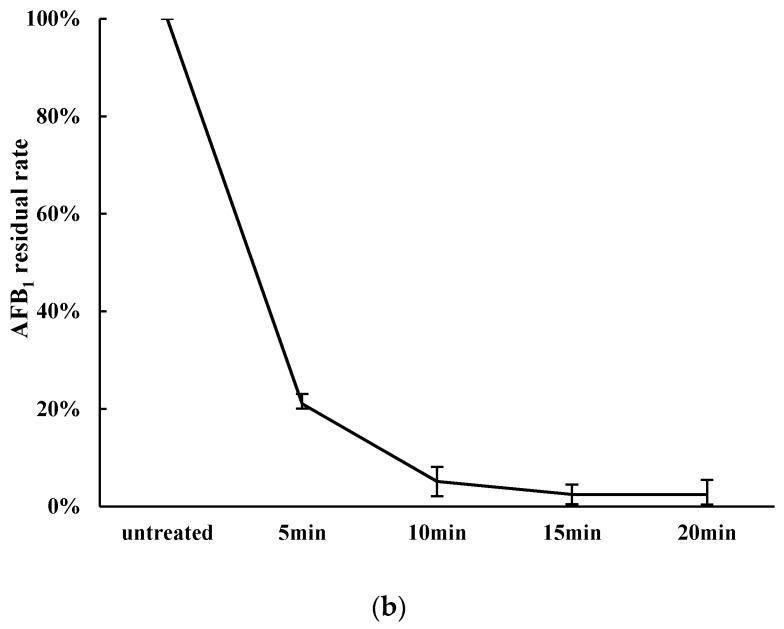
The effect of heat temperature and heating time on AFB_1_ detoxification by *Zygosaccharomyces rouxii*. (**a**) Influence of heating temperature on AFB_1_ detoxification. Peanut meal was fermented by 10 mL cell liquid after being heated for 10 min at pH 10; (**b**) Effect of heat time on AFB_1_ detoxification. Peanut meal was fermented by cell liquid after being heated at 100 °C at pH 10.

The effect of heating temperature on AFB_1_ decontamination is shown in Figure 1a. Ten grams of peanut meal was heated to 40, 60, 80, 100, or 110 °C for 10 min, and was then fermented by 10 mL cells liquid of *Z. rouxii* (approx. 1.0 × 10^9^ CFU/mL). The residual rates of AFB_1_ were 61.08%, 62.46%, and 49.63% after the peanut meal was heated at 80 °C, 100 °C, and 110 °C for 10 min. Increasing the heating temperature decreased the AFB_1_ content in peanut meal. After fermentation, the remaining AFB_1_ was decreased. The residual rates were 32.73%, 20.85%, 16.18%, 5.13%, and 5.10%, respectively after aerobic solid state fermentation by *Z. rouxii*. Above 100 °C, the reduction in AFB_1_ did not increase, so 100 °C was chosen as the optimal temperature in the experiment.

As shown in Figure 1b, heating time can also influence the detoxification of AFB_1_. Ten grams of peanut meal was heated at 100 °C for 5, 10, 15, or 20 min, and was then fermented by 10 mL cells liquid of *Z. rouxii* (approx. 1.0 × 10^9^ CFU/mL). The residual rates were 21.06%, 5.13%, 2.48%, and 2.44%, respectively, after aerobic solid state fermentation by *Z. rouxii* in peanut meal. When the heating time was 15 min, the residual rate of AFB_1_ reduced by 2.48%. As presented in Figure 1, with increasing heat temperature and heat time, the overall trend of detoxification rate was significantly increased. Thus, 100 °C and 15 min were chosen as the optimal condition in the experiment.

### 2.4. Removal of AFB_1_ in Liquid Fermentation

**Figure 2:**
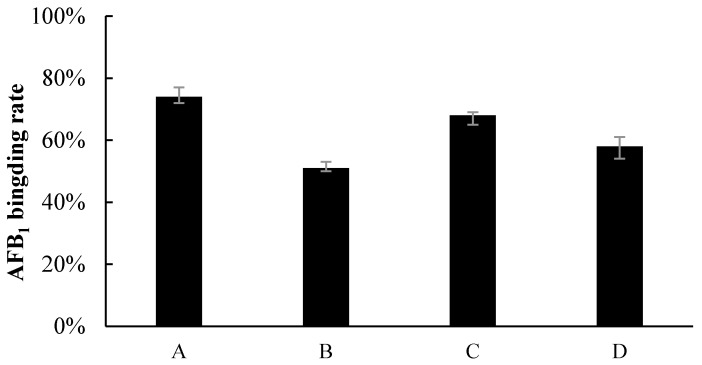
Percentage of AFB_1_ binding by (A) nonviable *Z. rouxii*, (B) viable *Z. rouxii*, (C) nonviable *S. thermophilus*, and (D) viable *S. thermophilus*.

The results showed that binding was the main effect of the removal of AFB_1_ in liquid fermentation (Figure 2). The amount of AFB_1_ bound by *Z. rouxii* and *S. thermophilus* was more than 50%. Specifically, the binding rates were 74.3% (nonviable cells of *Z. rouxii*), 51.4% (viable cells of *Z. rouxii*), 68.7% (nonviable cells of *S. thermophilus*), and 58.5% (viable cells of *S. thermophilus*), respectively. The binding effect was different between nonviable and viable cells. However, the supernatant of *Z. rouxii* and *S. thermophilus* could not lower the AFB_1_ concentration in the liquid, and this indicated that the cells of *Z. rouxii* and *S. thermophilus* could only remove aflatoxins by binding instead of degrading in liquid fermentation.

### 2.5. Verification of Detoxification of AFB_1_ by LC/MS

**Figure 3:**
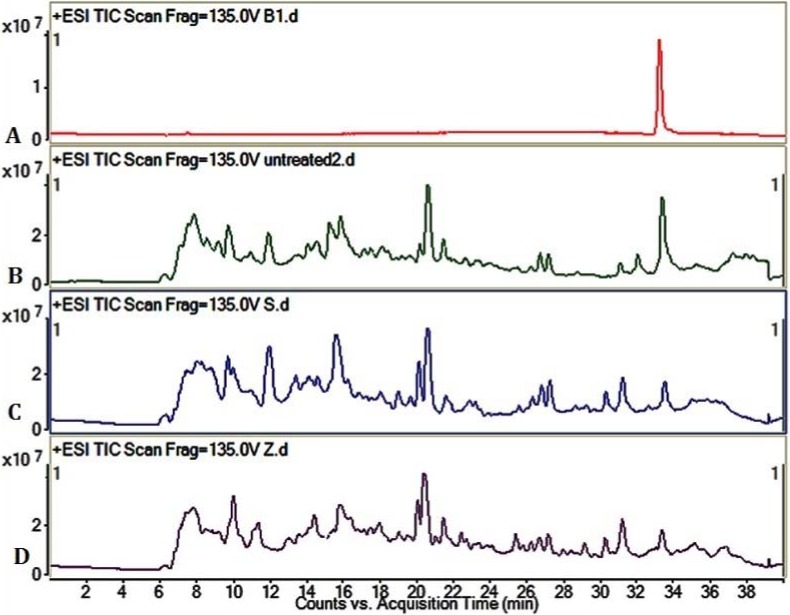
Liquid chromatography/mass spectrometry (LC/MS) profile of AFB_1_ degradation. (**A**) +ESI TIC scan of AFB_1_ standard substance; (**B**) +ESI TIC scan of untreated group; (**C**) +ESI TIC scan of *S. thermophilus* fermentation group. Peanut meal was fermented by *S. thermophilus* for 3 days at 37 °C; (**D**) +ESI TIC scan of *Z. rouxii* fermentation group. Peanut meal was fermented by *Z. rouxii* for 3 days in 28 °C. AFB_1_ of all groups was adequately dissolved and extracted by methanol.

The LC/MS profile of AFB_1_ degradation can be distinctly observed from the positive ESI TIC scan of AFB_1_ (Figure 3). The AFB_1_ standard substance group (a) indicated that the retention time of AFB_1_ was about 33.6 min. Compared with the untreated group (b), the AFB_1_ peaks in the *S. thermophilus* fermentation group (c) and *Z. rouxii* fermentation group (d) were obviously lowered, meaning that the AFB_1_ was effectively detoxified by solid state fermentation, which is consistent with HPLC results.

**Figure 4:**
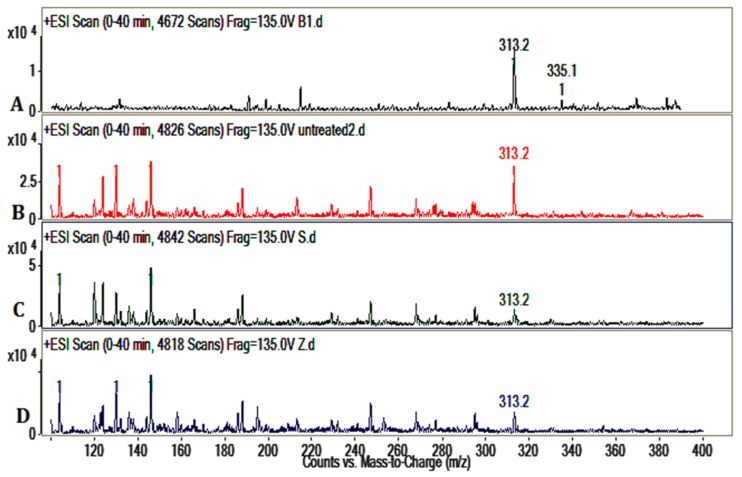
Detection of AFB_1_ by electron-spray mass spectrometry. (**A**) AFB_1_ LC/MS spectrum; (**B**) LC/MS spectrum of untreated group; (**C**) LC/MS spectrum of *S. thermophilus* group; (**D**) LC/MS spectrum of *Z. rouxii* group.

LC/MS spectrums of AFB_1_ showed the *m/z* 335, *m/z* 313 of AFB_1_ (Figure 4). As a comparison, the LC/MS results of the untreated group (b), *S. thermophilus* (c) group, and *Z. rouxii* (d) group revealed that AFB_1_ was detoxified and biotransformed to new substances whose structural properties were different from AFB_1_. This also demonstrated the complex compounds in peanut meal.

## 3. Discussion

In this study, there was no obvious reduction of AFB_1_ by cell-free liquid of *Z. rouxii* and *S. thermophilus*. We also tested the binding effects of *Z. rouxii* and *S. thermophilus* (Figure 2); the results showed that the two strains indeed had the ability to bind aflatoxins, as previous reported [24]. In peanut meal, however, AFB_1_ interacted tightly with three major protein fractions of peanut in a certain way [25], so AFB_1_ could not be scrambled by the cells of *Z. rouxii* or *S. thermophilus*. In other words, the removal modes of AFB_1_ in liquid fermentation and solid state fermentation are different. Although alkalinity–heat treatment could remove a certain amount of AFB_1_, when we combined alkalinity–heat and solid state fermentation of *Z. rouxii* or *S. thermophilus*, AFB_1_ could be almost completely removed. In Coomes’ report, in the process of alkalinity–heat treatment, humidity, alkalinity, and high temperature could hydrolyze the lactone ring of AFB_1_ to form *o*-coumaric acid, the characteristic bond became more active, and AFB_1_ became more easily degraded [25]. Studies reported that AFB_1_-degrading enzymes play an essential part in feed fermentation [26]; lactic acid bacteria and yeast metabolism could play important roles. Most of AFB_1_ was transformed into a new fluorescing compound corresponding to aflatoxin B_2a_ in acidogenous yoghurt [27]. Coincidentally, AFB_1_ was reduced during the fermentation of alcoholic beverages, and the degradation compound was also AFB_2a_ [28], which may be influenced by yeast fermentation. Therefore, we can further speculate that it is possible that the solid state fermentation of *Z. rouxii* and *S. thermophilus* have the possibility to biotransform AFB_1_ into harmless products. In the current study, we utilized LC/MS to analyze the feasible primary structure of degradation products. ESI TIC scan (Figure 3) and MS spectra (Figure 4) showed that the AFB_1_ peak of the untreated group was significantly higher than that of fermentation groups, but the figures did not reflect the presence of any new peaks. The TIC and LC/MS spectrums confirmed that AFB_1_ was degraded by *Z. rouxii* or *S. thermophilus*, which was also found by HPLC (Figure 1). AFB_1_ is most likely degraded to some new products whose structural properties were different from AFB_1_ [28,29]. Previous studies demonstrated that the unknown by-products dissolved in the aqueous phase rather than in the organic phase [1]; this may be the reason why we could not detect any degradation products in our study.

## 4. Conclusions

This study successfully screened out one strain of *Z. rouxii* that had the ability detoxify AFB_1_ with aerobic solid state fermentation in peanut meal. Through a single factor experiment of heating time and heating temperature, the optimal management condition of peanut meal was heating for 15 min at 100 °C, and the AFB_1_ residual rate was 2.48% after solid state fermentation by *Z. rouxii*. However, in liquid fermentation, cell-free liquid had no influence on AFB_1_ removal, and cells of *Z. rouxii* and *S. thermophilus* could indeed bind AFB_1_—especially nonviable cells of *Z. rouxii.* This means that AFB_1_ is removed by *Z. rouxii* through physical binding or biological degradation in liquid fermentation or in solid state fermentation, respectively. In addition, no degradation product was discovered from the LC/MS results. AFB_1_ may be degraded to some new products whose structural properties were different from AFB_1_, or they dissolved in the aqueous phase rather than in the organic phase.

## 5. Materials and Methods

### 5.1. Detoxification of Aflatoxins by Solid State Fermentation

#### 5.1.1. Screening Strains for Detoxification of AFB_1_

Traditional Chinese foods (fermented soy paste and yoghourt) are safe strain sources. A 1 g sample was put into a sterile tube, and 9 mL of sterile saline was added. After being fully shaken on a spiral device for 10 min, the liquid supernatant was diluted to 10^−5^, 10^−6^, 10^−7^, or 10^−8^ diluent, and then 20 μL diluent was spread onto the Yeast Extract Peptone Dextrose Medium (YEPD: 1% yeast extract, 2% peptone, 2% glucose, 2% agar). Finally, all samples were cultivated in an incubator at 30°C for 3 days. The colonies were singled out and inoculated to the YEPD agar. *S. cerevisiae* was purchased from Angel Company (Yicang, Hubei Province, China). *S. thermophilus* and *B. megaterium* were preserved in the School of Food Science and Engineering, Ocean University of China [19,28].

To test the AFB_1_ detoxification effect, *S. cerevisiae*, *S. thermophilus*, *B. megaterium*, and four strains from Chinese food (Table 1) were examined by solid state fermentation. Firstly, 10 g of peanut meal (Luhua Company, Laiyang, Shandong, China) and 10 mL deionized water (peanut meal:deionized water = 1:1, *w*:*v*) were placed in a 250 mL Erlenmeyer flask and the pH was adjusted to 10 with NaOH solution, then heated for 10 min at 80 °C. Secondly, the pH of the peanut meal was adjusted to 7 with HCl solution, and 10 mL precultured cells solution (approx. 10^9^ CFU/mL) was added in Erlenmeyer flask. Lastly, the peanut meal was fermented at 30 °C for 3 days. This experiment was repeated three times.

#### 5.1.2. Identification of Strain A

The isolated strains were identified by morphological, physiological, and chemotaxonomic features of the organism, and were confirmed by 26S rRNA basal phylogenetic analysis. Total genomic DNA as a template for PCR amplification was extracted by a Fungal Genomic DNA Isolation Kit (Sangon Biotech, Shanghai, China). The primers used for amplifying targeted conserved regions of the 26S rDNA gene were 5′-GCATATCAATAAGCGGAGGAAAAG-3′, 5′-GGTCCGTGTTTCAAGACGG-3′. EX-Taq DAN polymerase was purchased from Omega Bio-tek Company (Norcross, GA, USA). PCR was performed in an Applied Biosystems 2720 Thermal Cycler. The amplified PCR products were sent to Sangon Biotech for sequencing. The resulting sequence were submitted to NCBI [30] to compare with the published 26S rDNA sequences available in Genbank.

#### 5.1.3. Effect of the Heating Temperature and Heating Time on AFB_1_ Detoxification

Ten grams of peanut meal was heated at 40 °C, 60 °C, 80 °C, 100 °C, or 110 °C for 10 min, then inoculated with 10 mL precultured cells suspension (approx. 10^9^ CFU/mL) and fermented for two days. Next, the effect of heating time was investigated. Ten grams of peanut meal was heated at 100 °C for 5 min, 10 min, 15 min, or 20 min. Later, 10 mL precultured cells suspension (approx. 10^9^ CFU/mL) was inoculated in peanut meal. The residual content of AFB_1_ was determined by HPLC.

### 5.2. Removal of Aflatoxins by Cell and Cell-Free Liquid

To examine the ability to bind AFB_1_, 10 mL of precultured *Z. rouxii* or *S. thermophilus* suspension (approx. 10^9^ CFU/mL) was divided into two groups; the first group was viable cells, while the second group was boiled at 115 °C for 20 min (dead cells). Cells were centrifugated to pie at 5000 × *g* for 5 min. Cells was resuspended in 20 mL phosphate-buffered saline (PBS), and then 4 μL AFB_1_ (1 mg/mL) was added and cultured at 30 °C for 24 h and 48 h. As control, 4 μL AFB_1_ (1 mg/mL) was also added to the 20 mL PBS (without cells) and cultured at the same conditions. The bound percentage was calculated adhering to the formula: 100 × (1 − (amount of AFB_1_ in the supernatant/amount of AFB_1_ in the PBS control)).

In addition, to verify whether the fermentation supernatant had an influence on the removal of AFB_1_, 20 mL cell-free liquid was also cultivated with 4 μL AFB_1_ (1 mg/mL) for 24 h and 48 h at 30 °C. For comparison, YPD and MRS control groups were treated in the same way. The AFB_1_ concentrations of all samples were analyzed by HPLC.

### 5.3. Extraction of Residual AFB_1_ and HPLC Analysis

The fermented peanut meal (3 g) and NaCl (0.6 g) were weighed into a centrifuge tube. After adding 10 mL methanol/water (60:40, *v*/*v*) and homogeneous mixing for 10 min, the samples were centrifuged at 5000 × *g* for 20 min at room temperature. Five milliliters of supernatant was diluted with 5 mL ultrapure water. Next, 5 mL of the diluted sample was extracted in immunoaffinity columns (Huaan Magnech BioTech Co., Ltd., Beijing, China) and then eluted with 1 mL of methanol at a flow rate of about 1 drop per second. Then, the eluent was used to pre-column derivatization before they were quantified by HPLC analysis. The eluent was evaporated under a gentle stream of nitrogen at 45 °C up to dryness condition, and then it was derivatized with 200 μL n-hexane and 100 μL trifluoroacetic acid (TFA) for 15 min. After being evaporated to dryness again, the eluent was redissolved in 200 μL water–acetonitrile (85:15, *v*/*v*).

AFB_1_ standard substances were purchased from Sigma Chemical (St. Louis, MO, USA). AFB_1_ was analyzed according to retention time in the HPLC system. The samples were separated by HPLC equipped with a ZORBAX Eclipse XDB-C18 column (4.6 × 150 mm, 5 µm, Agilent, Palo Alto, CA, USA) and a 470 fluorescent detector (G1321A, Agilent, USA) (λexc 360 nm; λem 440 nm) using a mobile phase solvent of 10% acetonitrile, 40% methanol, and 50% water. The flow rate was 0.8 mL·min^−1^ and the injection volume was 20 μL.

### 5.4. Verification of Detoxification of AFB_1_ by LC/MS

To verify that the AFB_1_ was detoxified by *Z. rouxii* and *S. thermophilus*, 8 μL AFB_1_ (1 mg/mL) was added to 200 g peanut meal, and then fermented for at 30 °C for 3 days by *Z. rouxii* and *S. thermophilus* after alkalinity–heat treatment. Next, 300 mL methanol was added and shaken adequately for 3 h to extract aflatoxins. Peanut meal was filtered out with gauze. The methanol was dried to dryness and was then redissolved by 2 mL methanol. Finally, 1 mL sample solutions were waited for detection. The degradation products of AFB_1_ were determined by Agilent 6410 Triple Quad LC/MS coupled to a Agilent 1200 series HPLC (Agilent, Palo Alto, CA, USA) system. LC separation was implemented with a ZORBAX Eclipse XDB-C18 column (4.6 × 150 mm, 5 µm, Agilent, USA). The gradient elution method of LC/MS was applied as follows: (1) firstly, the column was eluted with acetonitrile-0.1% formic acid (5:95, *v*/*v*); (2) then the column was eluted for 20 min until the concentration of acetonitrile reached 40%; (3) the gradient elution was switched to acetonitrile-0.1% formic acid (60:40, *v*/*v*) in 5 min, and it was developed at 100% acetonitrile in 5 min and kept for 5 min; (4) the eluted ratio turned to acetonitrile-0.1% formic acid (5:95, *v*/*v*) again in 2 min, and kept for 3 min. Mass spectra were acquired within the range of *m/z* 100–500 in a positive ESI Scan analysis (Qualitative Analysis Mass Hunter software, Agilent, Palo Alto, CA, USA). The run time was 40 min with the flow rate of 0.2 mL/min.

## Figures and Tables

**Table 1:** Effect of different strains on aflatoxin B_1_ (AFB_1_) detoxification in peanut meal by solid state fermentation.

Group	Source	Management Methods	AFB_1_ Residual Rate (%) (Mean ± Standard Deviation)
Untreated			100
Strain A	fermented soy paste	80 °C, pH 10, 10 min	16.18 ± 0.2
Strain B	fermented soy paste	80 °C, pH 10, 10 min	36.68 ± 0.5
Strain C	fermented soy paste	80 °C, pH 10, 10 min	42.47 ± 0.1
Strain D	yoghourt	80 °C, pH 10, 10 min	37.67 ± 0.3
*S. cerevisiae*	Angel Company	80 °C, pH 10, 10 min	34.42 ± 0.5
*B. megaterium*	[23]	80 °C, pH 10, 10 min	36.46 ± 0.8
*S. thermophilus*	[21]	80 °C, pH 10, 10 min	17.63 ± 0.4
